# Embolic Strokes in Paroxysmal Atrial Fibrillation: Anticoagulation Failure or Something Else?

**DOI:** 10.7759/cureus.57741

**Published:** 2024-04-06

**Authors:** Muhammad Umer Riaz Gondal, John Lemoine, Muhammad Asad Hanif, Nayab Mirza, Shuaib Latif

**Affiliations:** 1 Internal Medicine, Reading Hospital, West Reading, USA; 2 Internal Medicine, Drexel University College of Medicine, West Reading, USA; 3 Cardiology, Reading Hospital, West Reading, USA; 4 Neurology, Reading Hospital, West Reading, USA

**Keywords:** pacemaker, transesophageal echocardiography, embolic stroke, anticoagulation failure, infective endocarditis

## Abstract

Infective endocarditis (IE) often presents with various signs and/or symptoms. However, at times, IE can present without outstanding clinical evidence but may carry devastating consequences if not detected and treated. We present a case of an 81-year-old female with paroxysmal atrial fibrillation who presented to the emergency department with slurred speech. Her National Institutes of Health Stroke Scale (NIHSS) score was one, and her physical examination was unremarkable. Brain imaging revealed bilateral multiple acute supratentorial and infratentorial infarcts. The patient was fully compliant on apixaban and had a dual-chamber pacemaker placed years earlier at an outside facility for unclear reasons. Although initially suspected to have experienced anticoagulation failure (ACF), transesophageal echocardiography (TEE) was ordered to evaluate for possible left atrial appendage closure procedure, which disclosed a mobile, echo-bright structure on the mitral valve consistent with IE. Blood cultures returned positive, the patient was treated with IV antibiotics, and apixaban was resumed. It can be challenging to suspect IE clinically, especially in deceptive or insidious cases with no signs/symptoms. Still, ACF is a diagnosis of exclusion, and all sources of embolic stroke (such as IE) must be thoroughly worked up before assuming treatment failure.

## Introduction

Infective endocarditis (IE) can have devastating consequences, such as stroke due to septic emboli. Stroke is often one of the presenting symptoms of endocarditis and can be the only manifestation [1]. Patients with embolic strokes should be evaluated for IE as it changes management. Neurologic complications are the presenting symptom in 20% of the cases, associated with poor prognosis (45% of deaths versus 24% in patients without these complications) [2]. IE usually presents with systemic symptoms such as fever and can have various physical examination findings.

We present a case of a patient with no signs or symptoms of infective endocarditis who had been on anticoagulation with apixaban and was initially thought to have anticoagulation failure, as embolic stroke is a feared consequence of atrial fibrillation. A thorough evaluation revealed vegetation on the mitral valve, leading to the diagnosis of infective endocarditis.

## Case presentation

An 81-year-old female with paroxysmal atrial fibrillation (on apixaban 5 mg twice a day) presented to the emergency department with slurred speech. She was hemodynamically stable, her National Institutes of Health Stroke Scale (NIHSS) score was one, and her physical examination was unremarkable. An EKG revealed sinus rhythm with a right bundle branch block. Her blood work, including CBC, comprehensive metabolic panel (CMP), lipid panel, and HbA1c, was unremarkable. An urgent CT brain revealed acute to subacute infarcts in the left cerebellar hemisphere. She was not a candidate for tissue plasminogen activator (tPA), as she was on anticoagulation. A CT angiogram of the head and neck was negative for large vessel occlusion, deeming her not a candidate for thrombectomy. A brain MRI showed multiple acute/subacute supratentorial and infratentorial infarcts with micro-hemorrhages in the left superior cerebellar vermis.

**Figure 1:**
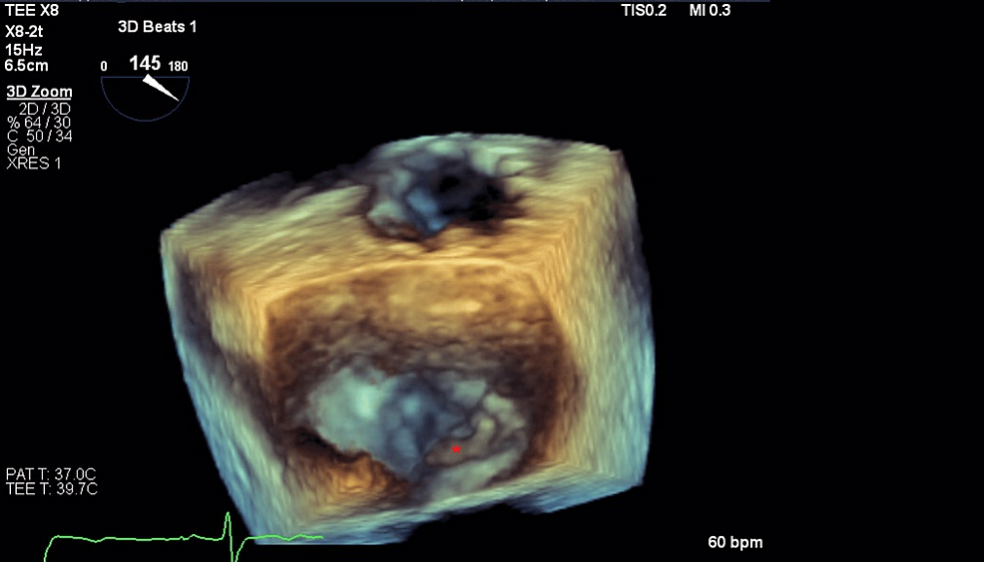
Three-dimensional (3D) image of TEE showing echo density on the posterior mitral valve leaflet (star). TEE: transesophageal echocardiogram

The patient had been fully compliant with her apixaban and had a dual-chamber pacemaker inserted at an outside facility a few years ago for unclear reasons. Cardiology was consulted for pacemaker interrogation, revealing a very low burden of atrial fibrillation, 0.2%. Initially, the cardioembolic strokes were believed to be due to apixaban failure, and the plan was for left atrial appendage closure and switching to warfarin. A transthoracic echocardiogram was unremarkable. A transesophageal echocardiography performed to evaluate appendage closure disclosed a mobile, echo-bright structure attached to the posterior mitral valve leaflet, likely representing ruptured chordae versus vegetation (Figure 1).

The TEE also showed trace mitral regurgitation with a negative bubble study and no thrombus. Given a benign physical examination and no risk factors, IE was initially low on the differential. Blood cultures were sent, which returned positive for *Streptococcus bovis*. The embolic strokes were then deemed secondary to IE. Her pacemaker was not removed as there was no evidence of vegetation on the pacemaker leads, and the patient was not septic. The patient was discharged with four weeks of IV ceftriaxone, with apixaban resumed after completion of treatment due to a high bleeding risk.

## Discussion

Atrial fibrillation is a common cardiac arrhythmia, seen in almost one in 10 Americans ≥80 years of age [3]. Stroke is the most frequent major complication faced by people with AF. Cardioembolic stroke secondary to AF occurs due to the delayed locomotion and increased turbulence of blood in the heart, leading to clot formation and eventual ejection to the systemic circulation. Patients with AF have a five-fold increased risk of stroke; about 1/3 of those with AF will have a stroke sometime in the course of their disease, and between 1/5th and 1/7th of strokes occur as a direct result of AF [4]. Thus, in addition to rate/rhythm control and management of co-morbidities, anticoagulation remains the mainstay of long-term AF management in reducing the likelihood of blood clot formation. Most patients with AF can benefit from anticoagulation. Still, the decision should be made individually considering patient preferences and scoring systems that stratify the risk of bleeding, such as the CHA2DS2-VASc score.

Despite adequate dosing and compliance with anticoagulation, some patients may still experience a stroke or other thromboembolic disease as a result of AF, a phenomenon known as anticoagulation failure (ACF). Rates of ACF occur at ~4% while receiving unfractionated or low molecular weight heparin and at 2-4% while receiving warfarin [5,6]. Rates of ACF in direct oral anticoagulants (DOACs), such as dabigatran, apixaban, and rivaroxaban have yet to be well-established. In suspected ACF, clinicians should first keep a broad differential diagnosis to determine if the thromboembolic event was due to the anticoagulant's failure versus medication non-compliance versus underlying thrombophilic condition such as malignancy or infection.

The patient in our case had been compliant with apixaban for paroxysmal AF when she presented with dysarthria and was found to have multiple acute/subacute infarcts on MRI. Due to the lack of other symptoms or signs of systemic illness, it was initially thought that she had undergone ACF. However, a thorough evaluation with TEE and blood cultures revealed the culprit causing her thrombophilic status - infective endocarditis (IE). Clinicians often rely on signs/symptoms listed in the 2023 Duke-ISCVID IE Criteria to warrant a workup for endocarditis [7]. General features in these criteria include fever, immunological phenomena, such as Osler's nodes and Roth's spots, and relevant cardiac risk factors (prior IE, presence of prosthetic valve, valvular heart disease). Although IE often presents these classical findings, many cases are insidious [8]. Prompt diagnosis and treatment are critical, as untreated IE can lead to several complications, such as heart failure, valvular regurgitation, or embolic events like the one seen in our patient.

Anticoagulation failure (ACF) is a diagnosis of exclusion and can only be made after extensive workup. Once medication non-compliance, as well as IE and thrombophilic conditions, have been ruled out, the clinician can declare ACF and must decide how to protect the patient from further thromboembolism. Generally, there are four approaches to managing ACF, but there needs to be more guidance on the most optimal solution [9]. The first option is dose escalation of previous medication. The BISTRO-II trial showed a significant dose-dependent decrease in thromboembolism with increasing doses of dabigatran [10], although later trials showed no reduction in mortality with increased doses of rivaroxaban [11].

The second option is switching to alternative medication. A meta-analysis proved that DOACs are generally preferable to warfarin in that they have lower rates of mortality, embolic stroke, and hemorrhagic stroke [12]. However, no head-to-head randomized controlled trials comparing the safety and efficacy of DOACs have been published. Additionally, the DOAC superiority was established on a population level; on an individual level, physicians may observe one anticoagulant as superior to another.

A third option is combining anticoagulants or adding an antiplatelet agent. The combination of anticoagulants with/without the addition of aspirin has been shown to reduce cardiovascular events in those with antiphospholipid syndrome [13]. Still, its value has not been established in cardioembolic stroke.

The fourth option is mechanical intervention. Devices such as inferior vena cava (IVC) filter and left atrial appendage (WatchmanTM) devices have been used in those who fail or cannot tolerate anticoagulation. The patient in our case was being evaluated for a left atrial appendage device for suspected ACF when she was incidentally discovered to have mitral vegetation, altering the diagnosis and management.

## Conclusions

Treatment failure is a legitimate risk in all medications, including anticoagulants. With the advent of novel therapies and the more widespread use of anticoagulants, rates of ACF may increase, and therefore, there will be a need for an evidence-based solution to manage ACF. However, when considering ACF in the differential, it is crucial to be thorough in the evaluation of other causes of thromboembolic stroke, such as insidious infective endocarditis. Our case highlights a deceptive case of infective endocarditis (IE) that led to the seeding of multiple acute/subacute cerebral infarcts in a patient otherwise compliant with anticoagulation for paroxysmal atrial fibrillation (AF). Therefore, for patients with AF who develop an embolic stroke, before considering the failure of anticoagulation, IE should be ruled out. In these patients with no signs or symptoms of IE and no predisposing risk factors, IE should still be one of the differential diagnoses.
